# Possible effects of left pulmonary artery stenting in single ventricle patients on bronchial area, lung volume and lung function

**DOI:** 10.3389/fped.2023.1337568

**Published:** 2024-01-15

**Authors:** Alessia Callegari, Julia Geiger, Fraser Maurice Callaghan, Christian Kellenberger, Jakob Usemann, Barbara Elisabeth Ursula Burkhardt, Oliver Kretschmar, Emanuela Valsangiacomo Büchel

**Affiliations:** ^1^ Pediatric Heart Center, University Children’s Hospital Zurich, Zurich, Switzerland; ^2^ Children’s Research Center, University Children’s Hospital Zurich, Zurich, Switzerland; ^3^ University Children’s Hospital Zurich, University of Zurich, Zurich, Switzerland; ^4^ Department of Diagnostic Imaging, University Children’s Hospital Zurich, Zurich, Switzerland; ^5^ Center for MR-Research, University Children’s Hospital Zurich, Zurich, Switzerland; ^6^ Division of Respiratory Medicine, University Children’s Hospital Zurich, Zurich, Switzerland

**Keywords:** single ventricle, Fontan, total cavopulmonary connection (TCPC), pulmonary artery stent, bronchial compression, lung volume

## Abstract

**Background:**

Left pulmonary artery (LPA) stenting is often required in single ventricle (SV) patients. Due to their close anatomical relationship an LPA stent could potentially compress the left main bronchus (LMB). We assessed the impact of LPA stenting on bronchial size, pulmonary volumes, and lung function in a cohort of SV patients.

**Methods:**

Forty-nine patients underwent cardiovascular magnetic resonance (CMR) and 36 spirometry 11 (8–15) years after Fontan. All patients were free of respiratory symptoms. LPA stents were inserted in 17 (35%) patients at 8.8 (3.4–12.6) years. Area/shape of the main bronchi (*n* = 46) and lung volumes (*n* = 47) were calculated from CMR-ZTE images for each lung and transformed in right-to-left (r/l) ratio and indexed for BSA. The effect of early stent insertion (prior to stage III) was analyzed.

**Results:**

Patients with LPA stent had larger r/l ratio for main bronchus area (*p* < 0.001) and r/l ratio difference for lung volumes was slightly larger in patients with early stenting. A trend toward a deformation of LMB shape in patients with LPA stent and toward a higher prevalence of abnormal spirometry in patients with early stent implantation was observed.

**Conclusions:**

In this cohort of patients, early insertion of LPA stents seems to relate with smaller LMB sizes and a trend toward smaller left lung volume and higher prevalence of impaired lung function. Whether these findings are caused by the stent or, at least to a certain degree, present prior to the implantation needs to be verified.

## Introduction

Surgical treatment of patients with a single ventricle (SV) consists of a staged palliation, which is considered completed after the Fontan operation (1). In the Fontan circulation, the systemic veins are directly connected to the pulmonary arteries, and the pulmonary blood flow circulation is passive, driven by the remaining post-capillary pressure and enhanced by inspiration (2). Over the last 50 years, continuous improvement in surgical, interventional, and medical approaches has significantly reduced the mortality of SV patients (3). At the same time, complications related to their unphysiological circulation have become more evident and remain a burden during mid- and long-term follow-up (1–3).

Adequate pulmonary artery growth and low pulmonary vascular resistance are major determinants of long-term outcome in Fontan patients (1). Stenosis of the central pulmonary arteries is frequent and leads to an obstruction and imbalance of pulmonary blood flow (4, 5). The left pulmonary artery (LPA) is mainly affected, as it may be compressed by the dilated (neo-)ascending aorta, or be hypoplastic due to a reduced blood flow (6–8). In these cases, the treatment of choice is percutaneous stent implantation, since it is minimally invasive, and the radial force of the stent permits to overcome external vessel compression (6, 7).

The left main bronchus (LMB) is anatomically located between the (neo-)ascending aorta and the LPA anteriorly and the descending aorta posteriorly. In case of stent implantation, ipsilateral bronchial compression or worsening of a pre-existing compression may occur due to the close proximity of these structures (9–11). Moreover, during follow-up, the stent needs to be re-dilated to match the patient's growth (4, 5, 12). In addition, restrictive ventilatory patterns have been described using lung function tests in these patients (13–16).

The aim of this study was to analyze the impact of LPA stenting not only on bronchial size, but also on pulmonary volumes and lung function. We hypothesized that: (a) patients with LPA stent have a relevant *bronchial compression*: the ratio of the area of the LMB to the right main bronchus (RMB) [right-to-left (r/l)] is increased in patients with LPA stent in comparison to those without LPA stent; (b) patients with LPA stent have a *smaller relative left lung volume*: the r/l ratio of the lung volume is increased, (c) a restrictive respiratory dysfunction is more frequent in patients with LPA stent in comparison to those without LPA stent; (d) patients with *early LPA stent implantation* have a significantly larger bronchial area r/l ratio and lung volume r/l ratio in comparison to those with later or no stent implantation.

## Methods

### Study design and patient selection

This is a single center, observational study (2019–2021) in a cohort of 49 Fontan patients (children and young adults). The patients were recruited from the cardiac electronic database of our institution. Inclusion criteria were ability to undergo CMR without the need for sedation (usually from school age), as well as written informed consent of the subject and his/her legal guardian. Exclusion criteria were younger age, any contraindication for CMR or pregnancy. Every examination was performed following the clinical standards of our institution. All examinations were performed within a maximal time interval of 4 months from each other.

LPA stents were implanted percutaneously. Early stent implantation was defined as any implantation before stage III (Fontan surgery). Patients with LPA stent implantation after stage III or no LPA stent implantation had similar bronchial area and similar lung volumes and were defined as a control group.

### Cardiovascular magnetic resonance

**Figure 1:**
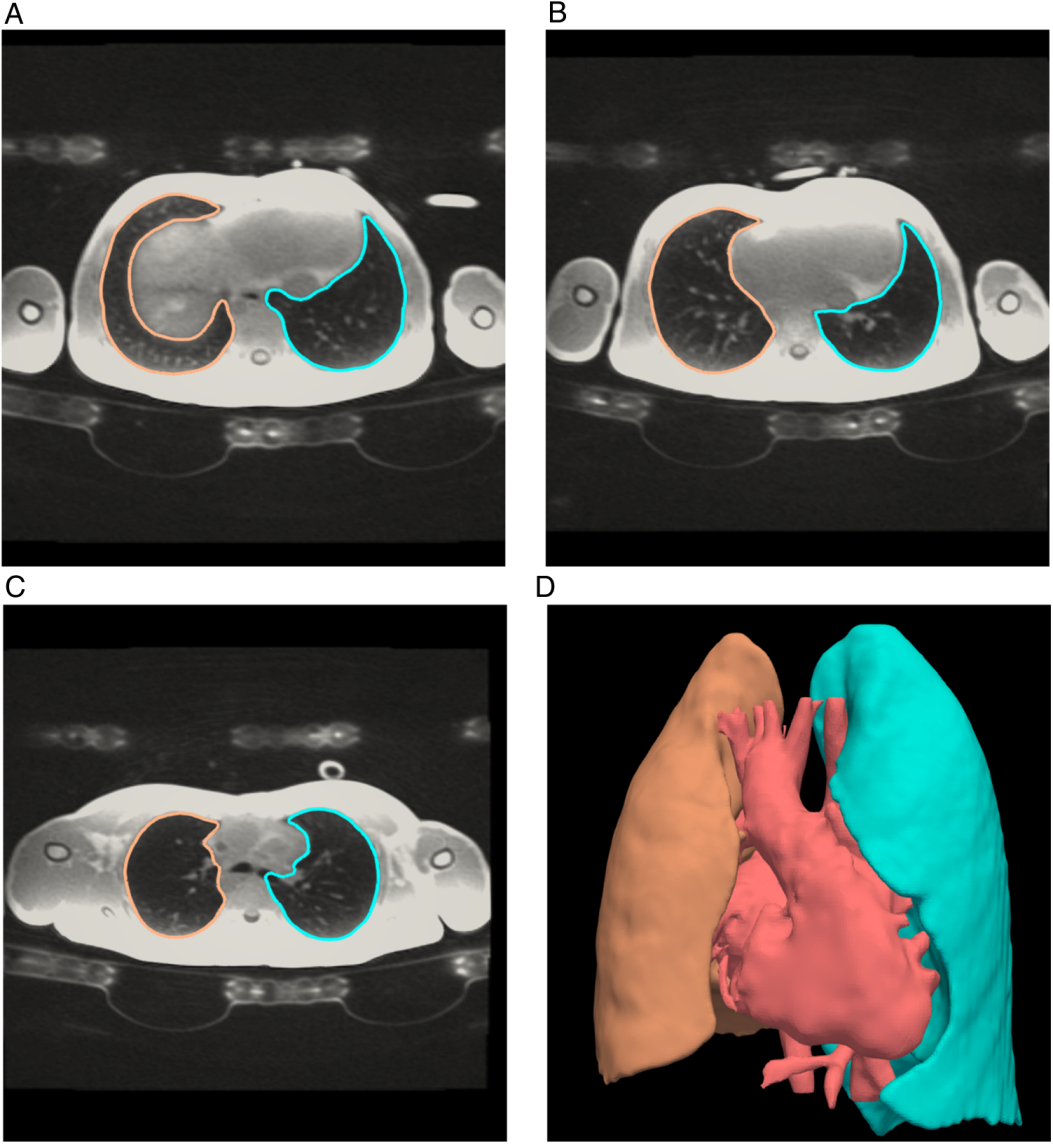
Lung volumes by magnetic resonance. Axial slices inferior to superior (**A–C**) from a 3D radial Zero TE CMR sequence of the lungs showing segmentation lines of left (blue) and right (orange) lungs. The 3D image (**D**) shows the complete lung segmentations as well as a rendering of the heart and major blood vessels. Segmentation was performed in a semi-automatic manner with manual quality control and adjustment where necessary. Lung volumes were calculated from the apex of the right and left lungs respectively and included lung vasculature.

All patients underwent cardiac magnetic resonance (CMR) examination in supine position on a 1.5 Tesla system (Discovery MR450 and Signa Artist, GE Healthcare, Waukesha, WI) using a 32-channel cardiac surface coil (GE Healthcare, Waukesha, WI). The MRI scanner was exchanged during the study period. Dedicated pulmonary imaging was performed in 47 patients using a 4D Zero echo time (ZTE) sequence in the first 22 subjects, a 3D ZTE sequence with respiratory triggering in the next 24, and an ultra-short echo time (UTE) radial cones sequence in one (17). MRI acquisition parameters for the lung imaging sequences are provided in Appendix 1. Lung segmentation was performed in a semi-automatic manner with manual quality control and adjustment where necessary. External bronchial segments were excluded from lung volume measurements, but intra-parenchymatous vasculature was included (Figure 1).

**Figure 2:**
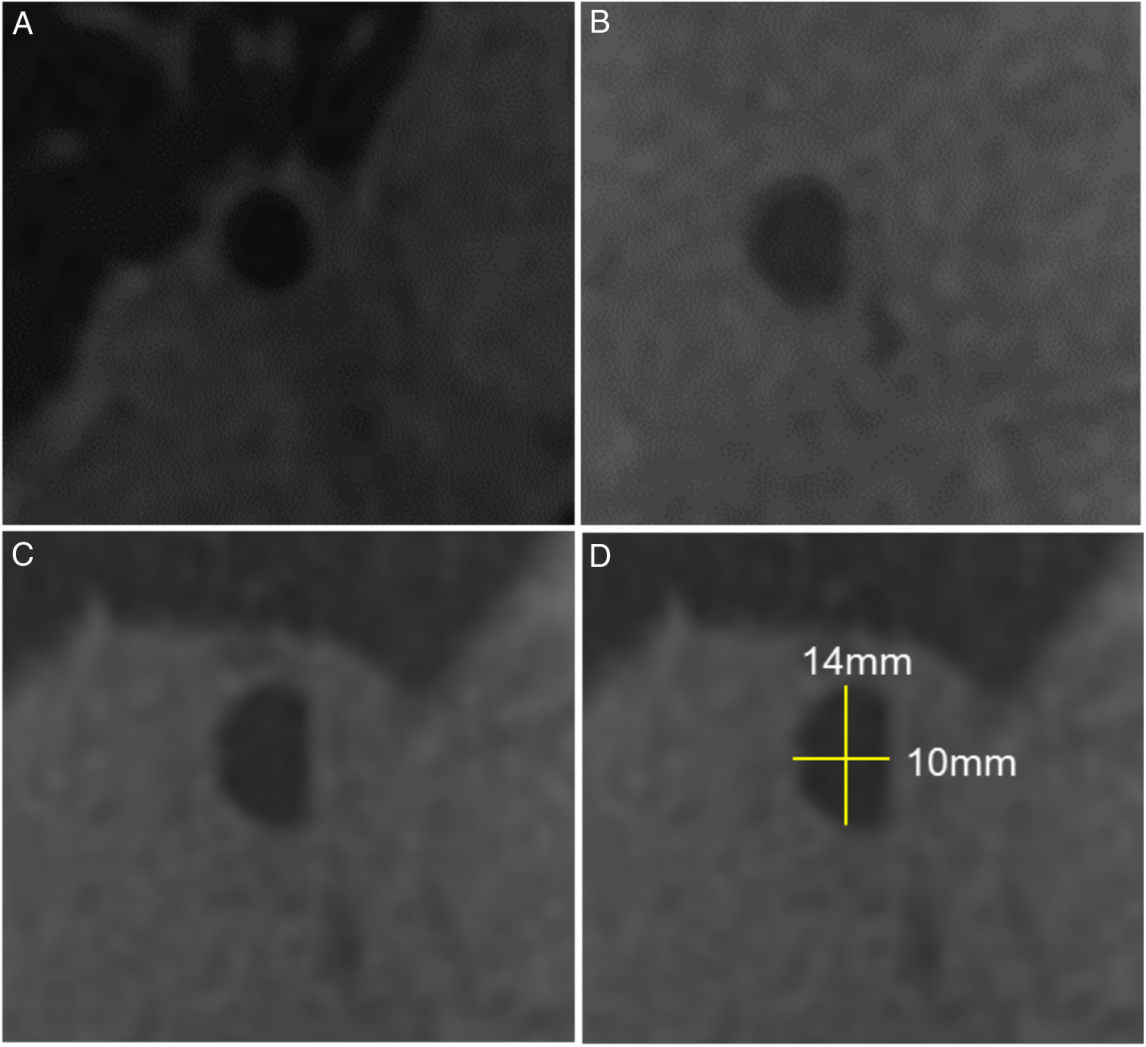
Bronchial diameters by magnetic resonance imaging. Examples of different bronchial shapes obtained from multiplanar reconstructions of the ZTE sequence: (**A**) round, (**B**) oval, (**C**) semilunar, (**D**) semilunar shape including diameter measurements.

Main bronchial diameters were measured on multiplanar reformatted images in the middle segment of the RMB and LMB between the carina and the segmental bronchial branching. All measurements were also indexed for body surface area and expressed as r/l ratio. The shape of the main bronchi was evaluated visually fand defined as oval, round or semilunar. The area was extrapolated with a geometrical formula by combining the diameters of the bronchi and their optical shapes. Examples of bronchial shapes and diameter measurements are shown in Figure 2.

### Spirometry

Spirometry tests were performed in 36 patients according to the European Respiratory Society/American Respiratory Society guidelines using Masterlab (Jaeger, Würzburg, Germany). Main parameters included the expiratory volume in 1 s (FEV1), forced vital capacity (FVC), and the FEV1/FVC ratio. We calculated *z*-scores using the Global Lung Function Initiative reference equations (18). Results with *z*-score <−1.64 were defined as abnormal test results.

### Statistics and ethics

Statistical analysis was performed using SPSS 27.0.0 (SPSS Inc, IBM Company, Chicago Illinois/USA). Continuous variables are expressed as median [interquartile ranges (IQR)], categorical data as counts (percentages). For normally distributed continuous variables, Levène's test for equality of variance was used to analyze if the variances in the two groups were significantly different, and group comparison was performed using independent two-sample *t*-tests. Kolmogorov-Smirnov analyses were used for group comparisons of non-normally distributed continuous variables. Ordinal, nominal, and dichotomous variables were evaluated with contingency tables and chi-square-tests. Significance was defined by values of *p* < 0.05.

The study followed the ethical guidelines of the declaration of Helsinki for medical research involving human subjects. The study was approved by the local ethical authorities (KEK ZH: 2018-01878).

## Results

### Patients and cardiac parameters

A total of 49 Fontan patients, 30 (61%) males, were included in the study. Median (IQR) age was 13 (11–16) years, weight 46 (35–59) kg, height 154 (142–166) cm, and body mass index 18.3 (17.1–21.2) kg/m^2^. Twenty-five (51%) patients had a single right ventricle, and hypoplastic left heart syndrome was the most frequent diagnosis (16 patients, 33%). Cardiac diagnoses are shown in Table 1. All patients had a left-sided descending aorta. Median age at Fontan surgery was 2.5 (2.2–2.9) years, and the time interval between Fontan surgery and the study exams was 11.8 (8.5–15.8) years. In all patients the Fontan technique consisted of an extracardiac tunnel. Cardiac medication was taken by 16 patients (33%) and consisted of an ACE inhibitor in 11 (22%), diuretics in 7 (14%), and sildenafil in 4 (8%). Anticoagulation consisted of aspirin in 44 (89%) and phenprocoumon in 5 (10%) patients.

**Table 1:** Patient characteristics.

Cardiac anatomy	*n*	%
Single left ventricle	24	49
Double inlet left ventricle	10	20
Tricuspid atresia	6	12
Pulmonary atresia with intact ventricular septum	5	10
Unbalanced atrioventricular septal defect	3	6
Single right ventricle	25	51
Hypoplastic left heart syndrome complex	16	32
Double outlet right ventricle	6	12
Unbalanced atrioventricular septal defect	3	6

### Stent implantation

**Figure 3:**
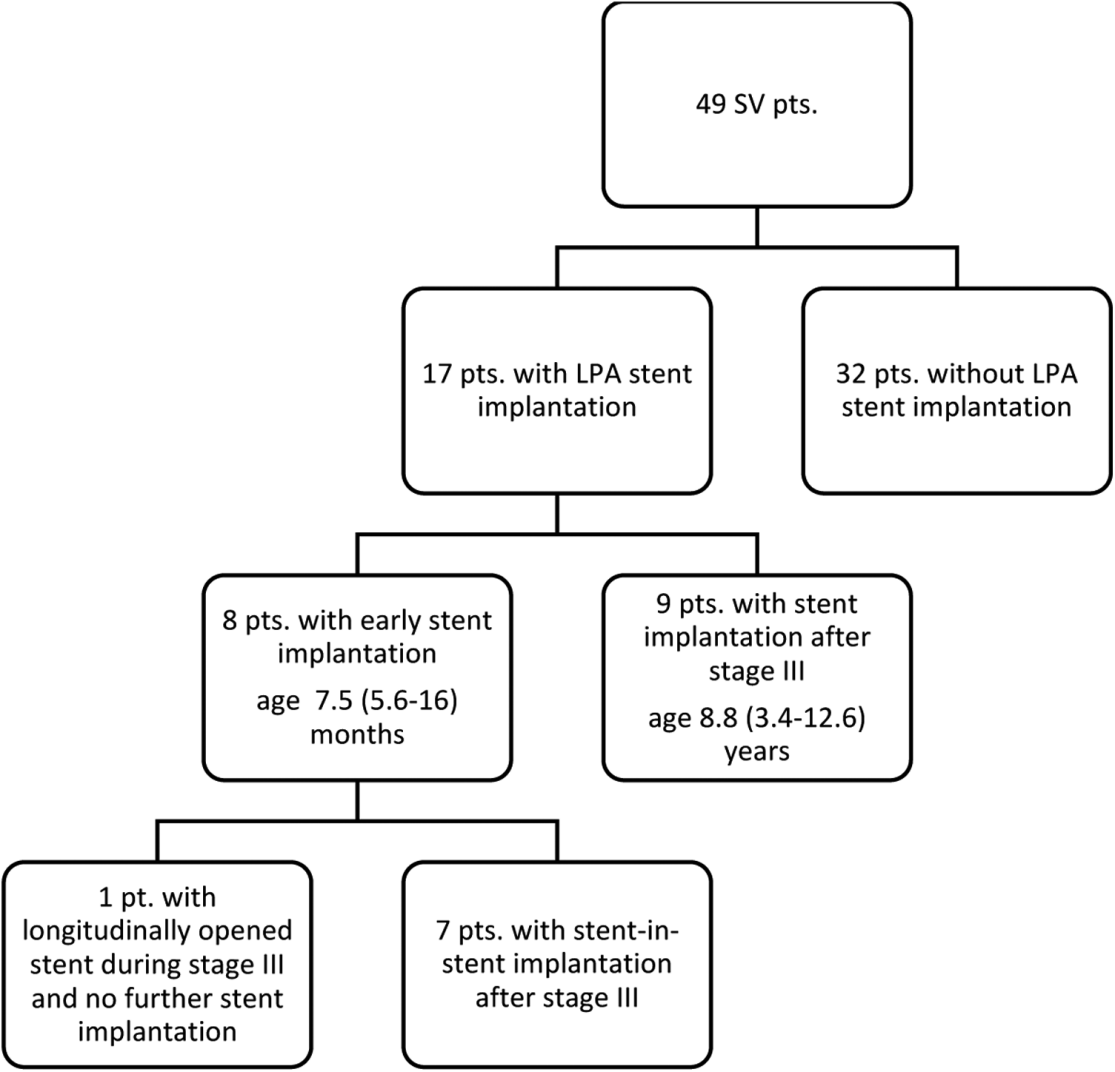
Flow chart of patients with/without stent. An LPA stent was implanted in a total of 17 patients (35%). Eight patients (47% of all stent patients) underwent early stent implantation, 7 of these underwent stent-in-stent implantation after stage III; while in one patient, the stent was partially longitudinally opened during stage III and no further stent was implanted. Nine further patients (53% of all stent patients) underwent late stent implantation after stage III.

LPA stenting was performed in a total of 17 patients (35%; Figure 3). Eight (47% of all stent patients) patients underwent early stent implantation at an age of 7.5 (5.6–16) months. At implantation, stent length was 15 (15–18) mm and diameter 6.5 (6–9) mm. All stents but one were re-dilated prior to Fontan surgery to a diameter of 8 (8–9) mm. Seven of these eight patients with early stent implantation underwent a stent-in-stent implantation after stage III while in one patient, the stent was partially longitudinally opened during stage III and no further stent was implanted. Nine further patients (53% of all stent patients) underwent stent implantation after stage III. After stage III, LPA stent implantation was at an age of 8.8 (3.4–12.6) years and stent diameter was 12 (10–16) mm and length 26 (21–35) mm.

All stents were pre-mounted, balloon expandable, bare metal stents with a closed cell or hybrid cell design (Cordis® Palmaz blue or Palmaz Genesis, or Cook® Formula, or Bentley BeGrow™ stents). No coronary stents were used.

### Bronchial area and shape

**Figure 4:**
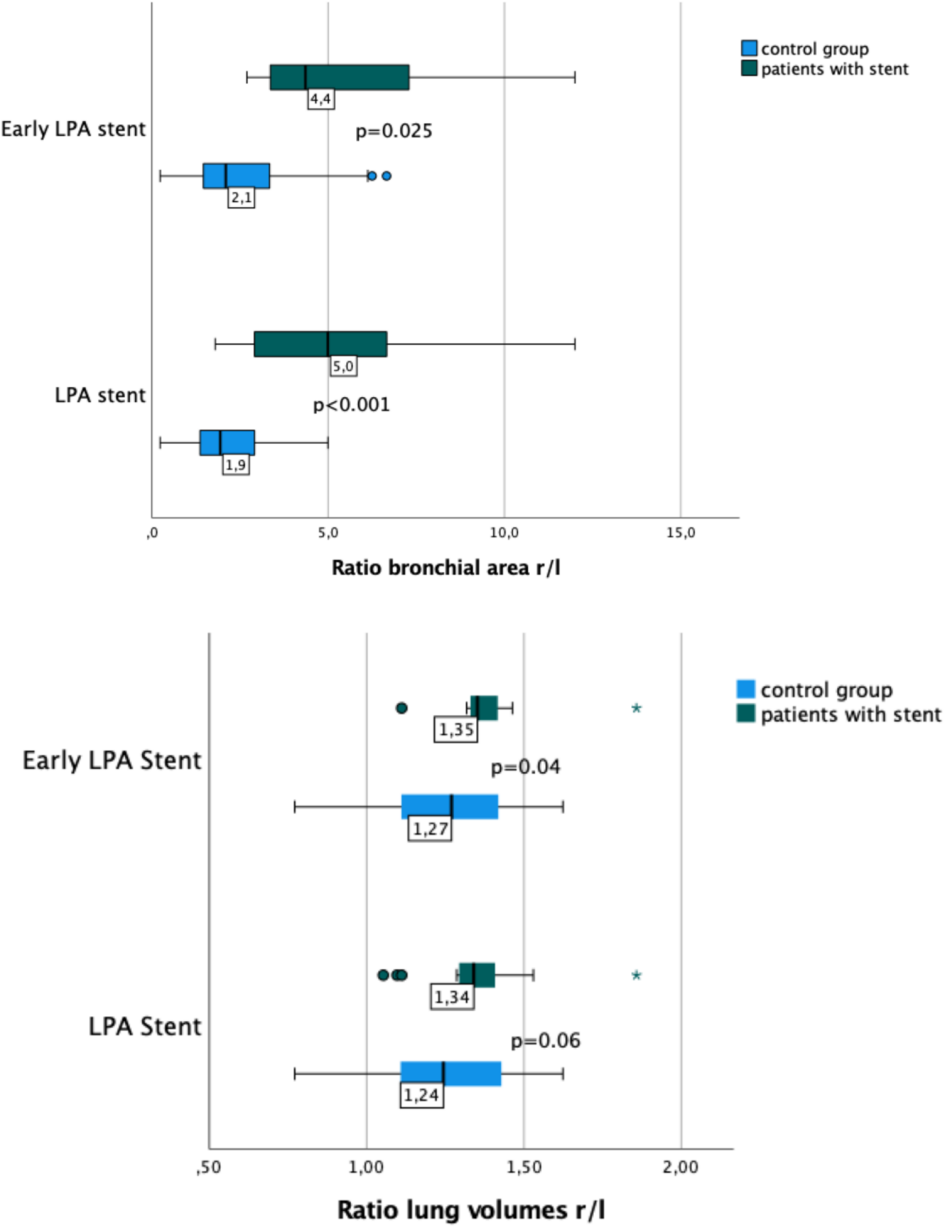
Box plots showing the ratio of right-to-left lung volume and bronchial area in patients with and without early LPA stent implantation. Patients with LPA stents had a larger r/l ratio for main bronchus area (*p* < 0.001) and a larger r/l ratio for lung volumes than patients without LPA stents. However, the difference in lung volume r/l ratio was only significant in patients with early LPA stenting (*p* = 0.04).

Bronchial area and shape could be assessed in 46 of 49 patients (Table 2 and Figures 2, 4). Two patients were excluded due to lack of lung imaging and one due to severe image artifacts.

**Table 2:** Bronchial area and shape.

	Total (*n* = 46)	LPA stent (*n* = 16)	No stent (*n* = 30)	*p*	Early LPA stent (*n* = 8)	No Early LPA stent (*n* = 38)	*p*
Area (mm^2^)							
Right	88 (69–104)	83 (66–101)	90 (6–108)	0.9	86 (66–86)	88 (69–105)	0.78
Left	32 (18–50)	**17** **(10–27)**	**42** **(27–53)**	**0** **.** **001**	**17** **(10–24)**	**36** **(26–52)**	**0** **.** **009**
Ratio r/l	2.5 (1.6–4.1)	**4.6** **(2.8–6.6)**	**2.0** **(1.4–2.9)**	**0** **.** **001**	**4.3** **(3.0–7.6)**	**2.0** **(1.4–3.4)**	**0** **.** **025**
Shape (right)							
Round	7 (15%)	4 (25%)	3 (10%)	0.8	1 (12%)	6 (16%)	0.79
Oval	26 (56%)	9 (56%)	17 (56%)	0.96	5 (63%)	21 (55%)	0.7
Semilunar	14 (30%)	4 (25%)	10 (33%)	0.55	2 (25%)	11 (29%)	0.97
Shape (left)							
Round	5 (11%)	3 (19%)	2 (7%)	0.26	1 (12%)	4 (11%)	0.87
Oval	35 (76%)	**9** **(56%)**	**26** **(87%)**	**0** **.** **002**	6 (75%)	29 (76%)	0.97
Semilunar	6 (13%)	4 (25%)	2 (7%)	0.07	1 (12%)	5 (13%)	0.98

The bold values are statistically significant.

In the overall group of patients with LPA stent, a smaller LMB area and a higher r/l ratio of the bronchial area were observed (*p* < 0.001). A similarly significant difference in both, bronchial area, and r/l ratio, was found comparing patients with early LPA stent implantation and patients with late implantation or no stent (Table 2). The most frequent bronchial shape—right and left—was oval. LPA stent presence caused a shift from an oval towards a more semilunar shape.

### Lung volume and surface

Lung volumes and lung surfaces were assessed in 47 of 49 patients. Two patients were excluded because of missing lung imaging. Lung volumes and lung surfaces were similar in patients with and without LPA stents (Table 3). Early LPA stent implantation also was not associated with any significant changes in surface area or r/l lung volume ratio (Figure 4), regardless of the *p* value obtained by statistical tests.

**Table 3:** Lung volume and surface.

	Total (*n* = 47)	LPA stent (*n* = 17)	No stent (*n* = 30)	*p*	Early LPA stent (*n* = 8)	No early LPA stent (*n* = 39)	*p*
Volume right lung							
ml	862 (616–1,187)	855 (616–1,223)	894 (612–1,194)	0.6	752 (668–1,249)	892 (601–1,187)	0.7
ml/m^2^	644 (550–722)	610 (534–755)	645 (552–726)	0.7	665 (571–837)	644 (518–722)	0.3
Volume left lung							
ml	691 (540–874)	622 (491–815)	728 (574–888)	0.6	595 (490–719)	736 (542–885)	0.2
ml/m^2^	512 (423–564)	471 (416–532)	523 (454–584)	0.2	481 (433–525)	519 (421–567)	0.7
Ratio r/l	1.3 (1.1–1.4)	1.3 (1.2–1.4)	1.2 (1.1–1.4)	0.1	**1.3** **(1.3–1.4)**	**1.2** **(1.1–1.4)**	**0** **.** **04**
Surface right lung							
cm^2^	643 (501–776)	611 (499–770)	647 (497–787)	0.6	558 (530–785)	648 (486–776)	0.4
cm^2^/m^2^	456 (417–504)	449 (426–511)	460 (403–501)	0.6	468 (441–572)	456 (391–500)	0.1
Surface left lung							
cm^2^	588 (475–687)	532 (455–643)	605 (486–704)	0.6	501 (450–602)	612 (478–700)	0.2
cm^2^/m^2^	407 (369–468)	394 (374–436)	419 (363–480)	0.3	402 (384–487)	417 (359–468)	0.7
Ratio r/l	1.1 (1.0–1.1)	1.1 (1.0–1.1)	1.0 (1.0–1.1)	0.06	1.14 (1.09–1.2)	1.0 (1.0–1.1)	0.1

The bold values are statistically significant.

### Lung function—spirometry

Spirometry was performed in 36 of 49 patients. FEV1 was abnormal (defined as *z*-score <1.64) in 13 patients (36%) and FVC in 12 (33%), indicating that both airway obstruction and restriction was present in approximately one third of the patients. Tiffeneau index was however normal 0.89 (0.85–0.93). No difference in prevalence of abnormal test results of FEV1 or FVC was observed in patients with or without LPA stent. Abnormal FEV1 (67% vs. 43%, *p* = 0.08) and abnormal FVC (67% vs. 39%, *p* = 0.07) tended to occur more frequently in patients with early LPA stent implantation.

## Discussion

In this study, we systematically assessed the impact of LPA stenting on bronchial area, lung volumes, and lung function in a cross-sectional cohort of Fontan patients. Our results show that early LPA stent insertion, i.e., implantation between stage II and III of Fontan palliation, seems to relate to smaller LMB area and a trend towards smaller ratio of left-to-right lung volume. Restrictive respiratory dysfunction was prevalent in all patients and especially in those with an early stent implantation.

We have recently investigated the reasons for LPA stenosis and found that patients with a single right ventricle and history of a Norwood I surgery required more frequently LPA stenting, and that the diameter of the reconstructed ascending aorta after DKS anastomosis was an independent risk factor (8).

As expected also in the specific cohort of this study, 13/17 patients of the stent group had a single right ventricle.

Extrinsic airway compression is a well-known complication directly after surgery and during long-term follow-up in patients after repair for congenital heart disease (19), particularly during staged palliation of single ventricle lesions (20, 21). Surgical and interventional procedures performed in a small thorax may affect the geometry of the intrathoracic structures. In many cases, the space between the reconstructed ascending aorta and the descending aorta is tight and may result in LPA and LMB compression (10). In our patient group, we observed that the LMB shape tends to become more semilunar than oval, which may be the result of a certain degree of stent-induced compression of the LMB.

The largest study on the prevalence of airway compression in patients with congenital heart disease was published by An et al. (19). They retrospectively analyzed computer tomography (CT) images of 2.729 patients (10% with a reconstructed aortic arch) and described airway compression in 58 (2.1%). Patients with aortic arch anomalies and vascular rings where found to be at higher risk; however the effect of endovascular stents on the adjacent airways was not assessed (22).

The first report of an ipsilateral LMB compression after LPA stenting was published in 2005 by Ferandos et al., who described the “mass effect” of PA stents on the adjacent structures with bronchial compression in 6 of 21 patients (29%) (23). All these patients had relevant respiratory symptoms that were assessed by computed tomography imaging (23). Since two patients died and one required tracheostomy, this study raised some concerns regarding critical clinical complications caused by LMB compression after LPA stent implantation. Our results are more reassuring, even though all our patients were free of symptoms and therefore may represent a different study collective.

In SV patients, airway compression may have even greater impact on Fontan circulation, as this is heavily dependent on a normal ventilation/perfusion relationship. Therefore, special precaution should be taken at time of LPA stent implantation (10, 11, 20, 24). Grohmann et al. (11) have described severe LMB obstruction in 2 of 19 SV patients after LPA stent implantation and successful treatment with balloon dilatation of the LMB. Others have suggested to simultaneously perform flexible bronchoscopy during stent insertion and inflation in order to prevent significant bronchial compression (9, 22). Pre-interventional risk stratification should be performed by using cross-sectional imaging (22, 23). More recently, Krings et al. (24) applied a novel double balloon technique in 11 SV patients to achieve an oval shaping of the LPA stent and prevent LMB compression.

As discussed above, genuine airway compression can occur in approximately 2% of all children with early surgery for CHD, independent of the presence of endovascular stents (19). In addition to the tight spatial conditions, in very young patients the airway tissue may still be soft and vulnerable to external compression. Our results support this hypothesis, as only patients with early stent insertion presented with a smaller bronchial area. Nevertheless, a relevant question remains unsolved; namely, is the growth of the bronchi being mainly affected by the presence of a stent or merely by diminished pulmonary blood flow? By using three-dimensional rotational angiography, Borik et al. (10) assessed anomalies of the pulmonary arteries and the airway in 25 children after SV palliation and found LMB stenoses in 10/12 children with concomitant LPA stenosis, even in the absence of pulmonary artery stents. These findings suggest that smaller bronchial areas may be present, at least to a certain degree, even before LPA stent implantation.

In the Fontan circulation, a preserved inspiratory function is more important than in other patients (16). In contrast to biventricular circulation, in Fontan patients, inspiration represents the main driving force for atrial and ventricular filling and therefore the most critical component for cardiac output at rest and during exercise, whereas myocardial contraction acts as a pump for forward flow (3, 16). A restrictive pattern of lung function is frequently found in Fontan patients (13, 14) and has been described to correlate with reduced exercise capacity and quality of life (15, 16). In our study, we found a similarly high prevalence of restrictive lung disease, with up to 33% of patients with pathologically diminished FVC. Moreover, our results show that early LPA stent implantation is related to smaller LMB and slightly smaller left lung volume, which potentially affect ventilatory function. Nevertheless, due to the small number of studied patients we were not able to statistically confirm this trend.

## Limitations

Our study was performed in a selected cohort of patients. Since we included only children able to undergo a CMR scan without sedation, younger patients, and patients with MR-incompatible devices could not be evaluated.

The control group was tailored specifically for our study, since CMR normal values for bronchial dimensions and lung volumes in Fontan patients do not exist. Thus, as we did not find any significant differences between patients without any stent and patients with late LPA stent implantation, we defined this group as the control group, and this could be a relevant bias in our analysis.

MR scanner exchange during the ongoing study with concomitant change of the lung imaging sequence, i.e., different type of ZTE, did not have an impact on bronchial delineation and lung volume quantification.

The cross-sectional design of the study does not provide any information about the longitudinal development of pulmonary dimensions, respiratory symptoms, and lung function. This may become subject of a further study.

## Conclusions

Percutaneous LPA stent implantation is an established procedure for relief of branch pulmonary artery stenosis in SV patients after Fontan. LPA stent insertion early during staged Fontan palliation seems to relate with smaller size of LMB and with a trend toward smaller left lung volume in relation to right lung volume. Early insertion of LPA stents might increase the prevalence of impaired lung function pattern in Fontan patients. Nevertheless, whether these findings are caused by the LPA stent implantation or, at least to a certain degree, are present prior to the implantation due to a combined vascular and air-way hypoplasia needs to be verified with further studies.

## Data Availability

The raw data supporting the conclusions of this article will be made available by the authors, without undue reservation.

## Appendix 1 MRI lung imaging sequence parameters.

**Table:**

	In plane resolution (mm)	Slice thickness (mm)	Echo time (ms)	Repetition time (ms)	Flip angle (degrees)
ZTE 4D (*n* = 22)	1.2 ± 0.08	1.5 ± 0.0	0.016 ± 0.0	327 ± 0.0	2
ZTE 3D (*n* = 24)	0.56 ± 0.03	2.0 ± 0.0	0.016 ± 0.0	573 ± 28	2
UTE Cones (*n* = 1)	0.91	3	0.032	3.7	5
